# Teleodontología en tiempos de la covid-19

**DOI:** 10.21142/2523-2754-0902-2021-062

**Published:** 2021-06-21

**Authors:** Paula Lucía Segura-Gaspar, Katherine Joselyn Atoche-Socola

**Affiliations:** 1 Carrera de Estomatología, Universidad Científica del Sur. Lima, Perú. lucia9711@hotmail.com Universidad Científica del Sur Carrera de Estomatología Universidad Científica del Sur Lima Peru lucia9711@hotmail.com; 2 División de Rehabilitación Oral, Universidad Científica del Sur. Lima, Perú. kattyas.22@gmail.com Universidad Científica del Sur División de Rehabilitación Oral Universidad Científica del Sur Lima Peru kattyas.22@gmail.com

**Keywords:** teleodontología, COVID-19, telesalud, videoconsulta, teledentistry, COVID-19, Telemedicine, videoconference

## Abstract

**Objetivo:**

Describir la utilidad de la teleodontología desde la llegada de la COVID-19 y cómo la virtualización puede beneficiar a los dentistas y pacientes en la consulta dental.

**Métodos:**

Se realizará la búsqueda bibliográfica utilizando las principales fuentes de datos de la literatura internacional en ciencias de la salud (Medline) utilizando las palabras “teleodontología”, “COVID-19”, “telesalud” y “videoconsulta”. La búsqueda se realizará sin restricción de idioma desde la fuente de información hasta el 30 de setiembre del 2020. Se analizarán tópicos, los cuales proporcionan información acerca de los diferentes beneficios de la teleodontología en tiempos de la COVID-19 y cómo se está utilizando en la consulta dental.

**Resultados:**

Se realizará el triaje de manera virtual por medio de videoconferencias, de manera que se evitará la propagación del virus. Se registrarán los datos del paciente en una ficha, en la cual se colocarán signos y síntomas del episodio actual del paciente, antecedentes médicos del paciente. En caso la emergencia o urgencia necesite ser tratada presencialmente, se deberá realizar un despistaje de COVID-19. El cuestionario puede realizarse por vía telefónica, y en él se preguntará sobre síntomas, antecedentes de viajes y contacto con algún paciente con COVID-19.

**Conclusiones:**

La teleodontología está siendo utilizada para asesorar, filtrar a pacientes con sintomatología y tratamientos de emergencia dentales.

## Abstract (en)

**Objective:**

To describe the usefulness of teledentistry during the COVID-19 pandemic and how virtualization can benefit dentists and patients.

**Methods:**

The bibliographic search will be carried out using the main data sources of international literature on health sciences (MEDLINE) databases up to September 30, 2020 with no language restriction. The information sought will include publications on the benefits in tele-dentistry during the COVID-19 pandemic using the search terms "Teledentistry, COVID-19, Telehealth, and video consultation”

**Results:**

Triage will be carried out virtually. Patient data, signs and symptoms of discomfort, medical history, among other variables. will be recorded. Cases of emergency or urgency will be treated in person, while a COVID-19 questionnaire including symptoms, travel history and contact with COVID-19 patients will be given by telephone.

**Conclusions:**

Teledentistry is being used for giving advice and to triage patients with symptoms and emergency treatments.

## INTRODUCCIÓN

A finales del 2019, en la ciudad de Wuhan, provincia de Hubei, en China, fueron diagnosticados 27 casos de neumonía de etiología desconocida ^1^, cuyo agente causal fue identificado en enero del 2020 a partir de muestras de frotis de la garganta que fueron analizadas por el Centro Chino para el Control y la Prevención de Enfermedades (CCDC). La nueva enfermedad fue nombrada COVID-19 por la Organización Mundial de la Salud (OMS) ^2^. El coronavirus que la provoca infecta principalmente el sistema respiratorio y puede transmitirse a través de gotitas respiratorias ^3^. Diversas investigaciones han demostrado que este virus puede transmitirse de una persona infectada a otra por medio directo, a través gotitas emitidas al toser o estornudar ^4^. Debido a esto la OMS, recomienda tomar estrictas medidas de prevención para evitar la transmisión a los cuidadores de pacientes con COVID-19 y en ambientes clínicos donde se producen aerosoles ^5^^,^^6^.

En la práctica dental se utilizan instrumentos rotatorios, raspadores ultrasónicos y jeringas triples que esparcen agua, saliva, sangre y otros desechos, durante el desarrollo de diferentes tratamientos de rutina. Estos aerosoles que flotan en el ambiente exponen a los profesionales dentales y a sus pacientes, de gran manera, a la transmisión de enfermedades por vía aérea ^7^. Dada la alta virulencia y difusión del virus, es importante reforzar las medidas de bioseguridad y añadir otras herramientas de trabajo para continuar con el control y la comunicación con los pacientes, y de esta manera reducir el número de visitas y el tiempo en la consulta dental ^8^.

En medio del estado de emergencia por la COVID-19, las consultas dentales se suspendieron, a fin de reducir la propagación del virus. En la actualidad, se están retomando las consultas o emergencias dentales de manera remota por medio de llamadas de telefónicas, correos, redes sociales, etc. ^9^. La pandemia ha llevado al personal de salud de las diferentes especialidades a pensar de una manera más ingeniosa, por ejemplo, la telesalud podría conservar la continuidad de la atención y evitar la difusión del virus ^10^. Los dentistas no son ajenos a esto y la están implementando. Por ello, el propósito de esta revisión será describir la utilidad de la teleodontología desde la llegada de la COVID-19 y cómo la virtualización puede beneficiar a los dentistas y los pacientes en la consulta dental.

## MÉTODOS

La búsqueda bibliográfica se realizó utilizando las principales fuentes de datos de la literatura internacional en ciencias de la salud: Medline vía PubMed, Scopus, ScienceDirect, EBSCO y LILACS. La búsqueda se realizó sin restricción de idioma desde la fuente de información hasta el 30 de setiembre del 2020. Se incluyeron revisiones sistemáticas y revisiones de la literatura. Se excluyeron cartas al editor, opiniones individuales y libros. Las estrategias de búsqueda incluyeron (Tabla 1) los términos teleodontología, COVID-19, telesalud y videoconsulta.

**Tabla 1:** Estrategias de búsquedas en las principales fuentes de información.

Estrategia para la búsqueda de artículos científicos en las principales fuentes de información	
Fuente	Términos de búsqueda
PubMed	((teledentistry) AND (covid.-19)) OR (e-dentistry),,,"(""teledentistry""[All Fields] AND (""severe acute respiratory syndrome coronavirus 2""[Supplementary Concept] OR ""severe acute respiratory syndrome coronavirus 2""[All Fields] OR ""ncov""[All Fields] OR ""2019 ncov""[All Fields] OR ""covid 19""[All Fields] OR ""sars cov 2""[All Fields] OR ((""coronavirus""[All Fields] OR ""cov""[All Fields]) AND 2019/11/01:3000/12/31[Date - Publication]))) OR ""e-dentistry""[All Fields]"
Scopus	teledentistry AND covid-19 OR e-dentistry OR videoconsultation AND (LIMIT-TO (PUBYEAR,2020) OR LIMIT-TO (PUBYEAR,2010)) AND (LIMIT-TO (DOCTYPE,"ar") OR LIMIT-TO (DOCTYPE,"re")) AND (LIMIT-TO (SUBJAREA,"DENT") OR LIMIT-TO (SUBJAREA,"MEDI"))
EBSCO	teledentistry AND (covid-19 or coronavirus or 2019-ncov or sars-cov-2 or cov-19) OR e-dentistry OR (video consultations or video appointments)
LILACS	tw:((tw:(teledentistry)) AND (tw:(covid-19)) OR (tw:(e-dentistry)) OR (tw:(videoconsulting))) AND (db:("LILACS") AND mj:("Telemedicina" OR "Consulta Remota" OR "Teleodontología")) AND (year_cluster:[2010 TO 2020])

### Telesalud

Este término se atribuye a los servicios de atención médica que hacen uso de tecnologías de la información y la comunicación a larga distancia como ayuda en la consulta, el diagnóstico, la planificación del tratamiento, la programación y el autocuidado. Las herramientas tecnológicas comprenden internet, videoconferencias, almacenamiento y retransmisión de imágenes ^11^.

### Teleodontología

Fue planteada por el ejército estadounidense en 1994, para servir a las tropas estadounidenses en el mundo, aumentar el acceso de los pacientes a una atención odontológica de calidad y establecer un sistema de telemedicina beneficioso ^12^^,^^13^. Durante la pandemia, ha sido utilizada como una opción en caso urgencias o emergencias dentales, y para ayudar a los pacientes a controlar sus tratamientos de manera remota. 

### Triaje

Recopila datos para determinar el tipo de atención estomatológica. Se realiza por teléfono o videoconferencia, con el fin de conocer signos y síntomas del paciente. Este paso es de suma importancia, ya que nos permite tener interacción con el paciente, ya sea por una videoconferencia o por teléfono, para recopilar los antecedentes y el motivo de consulta, y definir, en los casos en los que se requiera, el apoyo de diferentes especialidades. La atención presencial prioriza los casos de emergencias o urgencias estomatológicas. La Asociación Dental Americana (ADA) considera las emergencias dentales como situaciones que requieren tratamiento inmediato para detener el sangrado tisular en curso o para aliviar el dolor severo o la infección, que son potencialmente mortales ^14^ (Tabla 2).

**Table:** 

Emergencias dentales
• Sangrado descontrolado • Celulitis o infección bacteriana difusa de tejidos blandos, con hinchazón intraoral o extraoral que potencialmente compromete las vías respiratorias del paciente • Trauma que involucre huesos faciales y que potencialmente comprometa las vías respiratorias del paciente
Tratamientos de atención dental urgente
• Dolor dental severo por inflamación pulpar • Pericoronitis o dolor del tercer molar • Osteítis posoperatoria quirúrgica o cambios de apósito para alveolitis seca • Absceso o infección bacteriana localizada que produce dolor e hinchazón localizados • Fractura de diente que provoca dolor o traumatismo de tejidos blandos • Traumatismo dental con avulsión/luxación • Cementación del tratamiento dental si la restauración temporal se pierde, se rompe o causa irritación gingival

Debido a que las medidas de bioseguridad están siendo reforzadas con más barreras de bioseguridad durante la pandemia, como la mascarilla facial, el protector facial y los mandilones, es complicado que los pacientes puedan visualizar nuestros gestos al momento de la consulta, los cuales son de suma importancia porque generan cierta calma y seguridad a los pacientes. Esta primera consulta virtual nos permite interactuar con el paciente como si hubiese sido una consulta dental presencial prepandemia, ya que este es capaz de conocer a su odontólogo cara a cara, lo que contribuiría a generar un ambiente seguro durante la consulta. Por ello, es importante que el dentista mantenga una comunicación eficiente, maneje su tono de voz, transmita confianza y empatía hacia su paciente y cuidadores. 

### Consulta virtual 

Se requerirá llenar una ficha en la cual se registrarán signos y síntomas del episodio actual del paciente, antecedentes médicos y, ya que no es posible realizar un examen físico, se podrá llegar a un diagnóstico presuntivo y brindar al paciente orientación terapéutica. El odontólogo decidirá si es necesaria una consulta presencial para confirmar un diagnóstico presuntivo ^15^.

### Consulta presencial

Los odontólogos deberán realizar un despistaje de COVID-19; para ello, se puede realizar un cuestionario por vía telefónica, mediante el cual se consultará sobre síntomas, antecedentes de viajes y contacto con algún paciente con COVID-19 (Tabla 3). Además, Se deben aplicar todas las medidas de bioseguridad y se realizará recomendaciones al paciente para la atención estomatológica a fin de minimizar las vías de contagio ^(16, 17)^ (Tabla 4).

**Tabla 3:** Cuestionario telefónico recomendado a los pacientes que acuden a su cita odontológica.

1. ¿Tiene fiebre o la ha tenido en los últimos 14 días?
2. ¿Ha tenido problemas respiratorios (incluyendo tos) en los últimos 14 días?
3. ¿Ha viajado fuera del país en los últimos 14 días?
4. ¿Ha estado en contacto con alguna persona con confirmación o sospecha de tener COVID-19?
5. ¿Ha estado en contacto estrecho, en los últimos 14 días, con personas que presentaban un cuadro respiratorio agudo?

**Tabla 4:** Recomendaciones para el paciente al acudir a su cita odontológica.

• Cumplir con el horario estipulado de la cita con el fin de evitar la aglomeración de pacientes en el establecimiento de salud.
• No acudir acompañado, salvo que sea menor de edad o requiera asistencia de una persona.
• Acudir al establecimiento de salud con mascarilla.
• Al llegar al establecimiento de salud, lavarse las manos con agua y jabón durante 20 segundos o hacer uso de alcohol en gel.
• Mantener 1 metro o más de distancia con toda persona que se encuentre en el establecimiento de salud.
• Se priorizarán los tratamientos de emergencia o urgencia estomatológica.

### Videoconsultas

La interacción doctor-paciente es un punto muy importante, dada la situación actual del mundo, esto se ve limitado ya que implica un riesgo de trasmitir el virus. El campo de la salud en el mundo ha implementado la videoconferencia para reforzar las consultas de los pacientes. A pesar de que las consultas por videoconferencia en tiempo real son superiores a las realizadas por teléfono, no se espera que sustituyan a las interacciones cara a cara ^18^^,^^19^. Esta forma de atención acepta el uso de tecnología como teléfonos, teléfonos inteligentes o computadoras que, por medio de un enlace compartido, posibilitan la comunicación por video ^20^. Además, permiten al profesional en salud conectar con otros especialistas para tomar decisiones o referirlos a las diferentes especialidades, y de esta manera brindar un mejor servicio y responder a las necesidades del paciente ^21^. Las consultas virtuales pueden ser de gran ayuda para detectar, filtrar pacientes con sospecha de COVID-19, tratar síntomas y calmar a pacientes que presenten ansiedad dental ^22^.

### Limitaciones

Un examen completo incluye la exploración física de tejidos blandos y duros, lo que se ve limitado durante las consultas virtuales. Por otro lado, las fotografías enviadas por los pacientes pueden presentar una resolución baja, lo que dificulta el análisis ^23^. Además, el uso de la teleodontología por parte de profesionales que no conocen o se les dificulta el manejo de la tecnología puede causar cierto rechazo respecto de su adopción ^24^.

El acceso a internet es crucial en el uso de teleodontología, por lo que en ciertas zonas rurales el acceso es imposible. Además, la señal de internet debe ser adecuada para lograr una videoconsulta sin interrupciones. Para algunos pacientes, en su mayoría adultos mayores, el manejo de la tecnología es difícil por la falta de familiaridad, lo que genera ansiedad y, muchas veces, rechazo hacia esta herramienta ^25^.

## DISCUSIÓN

Los diferentes gobiernos alrededor del mundo tuvieron que tomar medidas importantes de restricción a la salida de las personas, para evitar el contagio. La teleodontología, mediante conferencias virtuales, ha sido de gran utilidad en el manejo a distancia de pacientes quirúrgicos y no quirúrgicos, al reducir costos, tiempo de espera de pacientes y propagación del virus ^26^^,^^27^. 

Una de las limitaciones de la teleodontología es la falta de servicios por tele salud, por lo que es importante el reentrenamiento del personal para capacitarlo en la atención del paciente en una consulta remota ^28^. Se han presentado casos de profesionales odontólogos que no se sentían cómodos al hacer un diagnóstico utilizando imágenes entregadas por telecomunicaciones sin información clínica ^29^. Sin embargo, los pacientes también necesitan contar con dispositivos para mantener las videoconferencias. Siendo así, es importante tener en cuenta que desarrollar un programa eficaz de telesalud lleva tiempo, por lo que es necesaria la educación y la capacitación de los profesionales para hacer frente a esta nueva realidad ^30^.

En una encuesta realizada a profesionales dentistas australianos sobre teleodontología, la mayoría coincidió en que es útil para el manejo del paciente y con el fin de aumentar la satisfacción respecto de los tratamientos, aunque un gran número refirió cierta incertidumbre con relación a la confiabilidad del método, la privacidad, los gastos de la práctica, el costo de instaurar la teleodontología y la precisión del diagnóstico ^31^. Más allá, es necesario que los servicios de teleodontología sean reconocidos por organismos reguladores y aseguradoras, con la finalidad de motivar a los profesionales dentales a utilizar la prestación de atención dental y mejorar la accesibilidad de los pacientes ^32^.

Finalmente, los odontólogos deben aceptar las limitaciones de la teleodontología y explicar a los pacientes que, en ciertos casos, se requerirá una consulta presencial para realizar un mejor diagnóstico. Por lo tanto, es importante que se sigan desarrollando los protocolos y procedimientos en lo que corresponde a la documentación y al consentimiento informado, así como el asegurar la privacidad y la confidencialidad de la información personal ^33^.

## CONCLUSIÓN

En tiempos de pandemia, la asistencia a centros de salud para consultas dentales es un riesgo, por lo que los profesionales dentales se han adaptado a la nueva realidad y han adaptado sus prácticas de la mano de la tecnología, a fin de dar continuidad a los controles de sus pacientes y de atender emergencias dentales. La teleodontología viene siendo utilizada para asesorar, filtrar a pacientes con sintomatología y brindar tratamientos para emergencias dentales.

## Fuente de financiamiento:

Autofinanciado
